# Supplementary material for: Polη O-GlcNAcylation governs genome integrity during translesion DNA synthesis
Source: Nat Commun. 2017 Dec 5;8:1941. doi: 10.1038/s41467-017-02164-1 (PMC5717138; doi:10.1038/s41467-017-02164-1)
Supplement: Supplementary file 1 — Supplementary Information [file 41467_2017_2164_MOESM1_ESM.pdf]

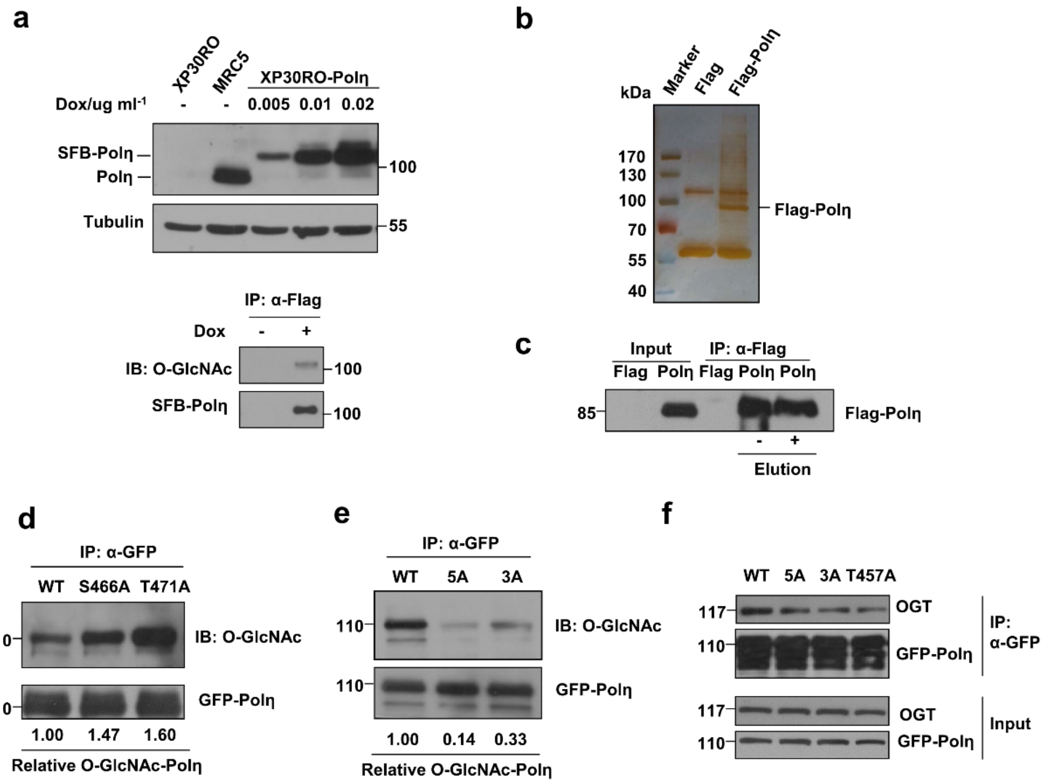

Supplementary Figure 1. Mapping the potential O-GlcNAcylated residue(s) in Polη. (**a**) XP30RO-Polη cells were treated with the indicated concentrations of Doxycycline (Dox) for 14 h. The cell lysates were harvested and blotted with Polη and tubulin antibodies (Top panel). XP30RO-Polη cells treated with doxycycline (0.01 μg ml^{-1}) were lysed and immunoprecipitated with anti-Flag M2 beads followed by immunoblotting with O-GlcNAc and Flag antibodies (Bottom panel). (**b**) & (**c**) 293T cells were transfected with Flag-Polη or empty vector, followed by treatment with Thiamet-G and glucose. The cell lysates were immunoprecipitated with anti-Flag agarose. (**b**) The immunoprecipitates were separated on SDS-PAGE and the expected Polη band was cut out for in-gel digestion followed by HCD-MS analysis. (**c**) The glycine eluted immunoprecipitates were examined by immunoblotting with anti-Flag antibody. (**d**) & (**e**) 293T cells transfected with WT and mutated GFP-Polη constructs were treated as in (**b**). The cell lysates were immunoprecipitated with anti-GFP agarose, followed by immunoblotting with anti-O-GlcNAc and anti-GFP antibodies. (**f**) 293T cells were transfected with WT or mutated GFP-Polη constructs. The cell lysates were immunoprecipitated with anti-Flag agarose, followed by immunobloting with anti-OGT and anti-Flag antibodies.

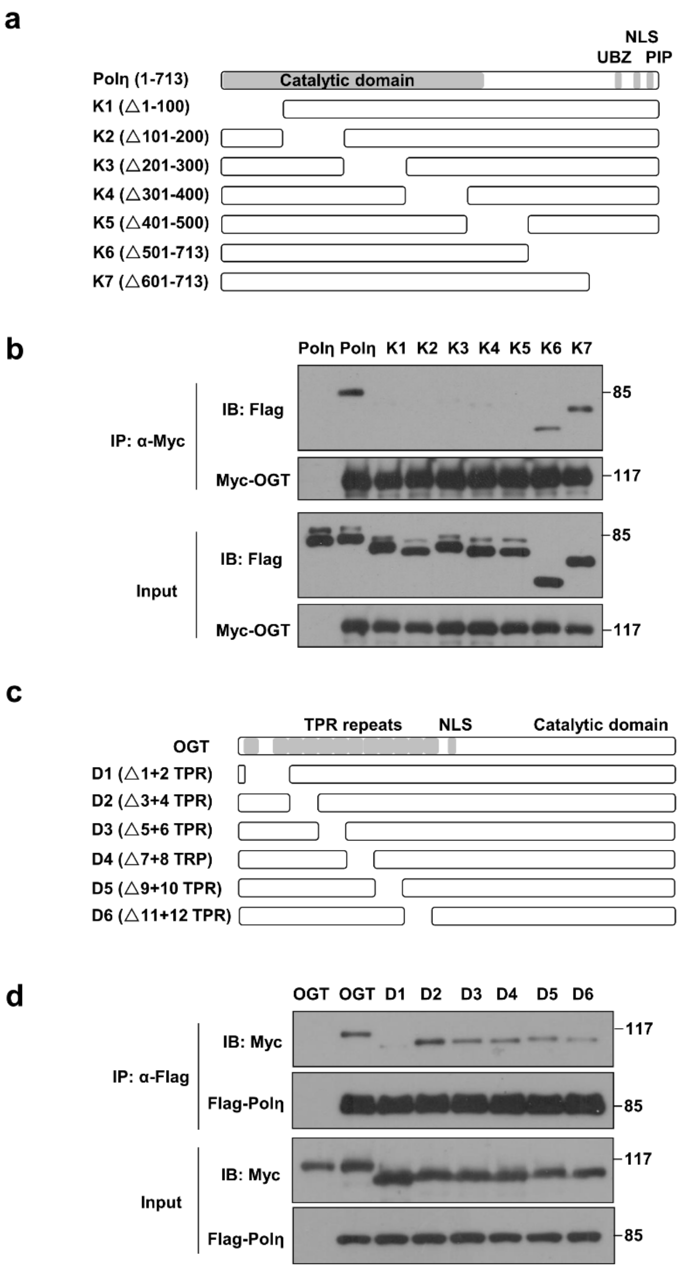

Supplementary Figure 2. Mapping the binding domains in Polη and OGT mediating their interaction. (**a**) Schematic representation of full-length and internal deletion mutants of Polη constructs. UBZ: ubiquitin binding zinc finger. NLS: nuclear localization signal. PIP: PCNA interaction peptide. (**b**) The full-length and deletion mutants of Polη were co-expressed with Myc-OGT in 293T cells. The cell lysates were immunoprecipitated with anti-Myc agarose beads followed by immunoblotting with Myc and Flag antibodies. (**c**) Schematic representation of full-length and internal deletion mutants of OGT constructs. (**d**) The full-length and deletion mutants of OGT were co-expressed with Flag-Polη in 293T

cells. The cell lysates were immunoprecipitated with anti-Flag M2 beads followed by immunoblotting with Myc and Flag antibodies.

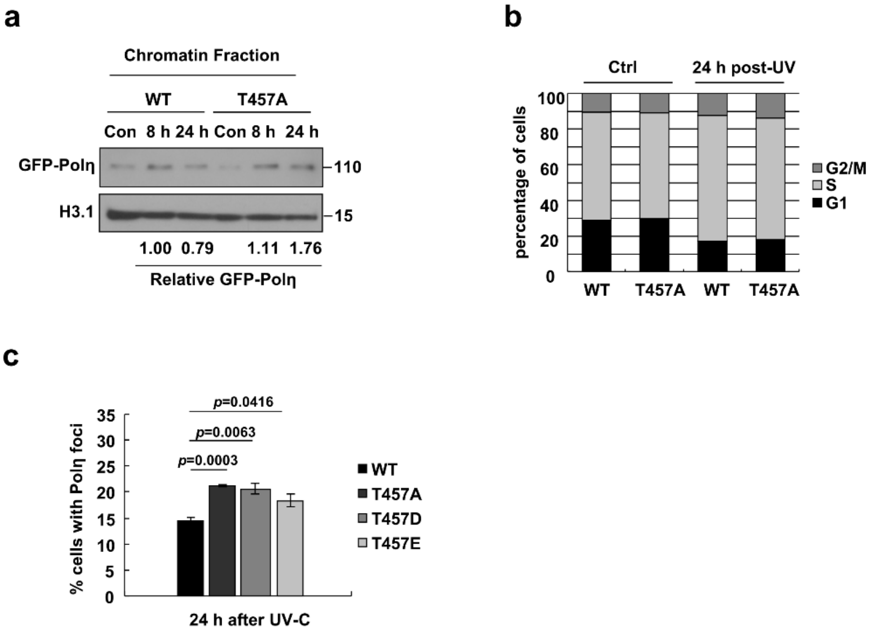

Supplementary Figure 3. Impaired T457A Polη chromatin removal post-UV was not due to change in cell cycle profiles or loss of phosphorylation at T457. (**a**) XP30RO cells stably expressing WT or T457A GFP-Polη were irradiated with UVC (15 J m^{-2}) and harvested at the indicated time points. The chromatin fractions were extracted followed by immunoblotting with Polη and H3.1 antibodies. (**b**) Cell cycle distribution of cells expressing WT or T457A in Fig. 3**a** is determined by flow cytometry. (**c**) U2OS cells transfected with WT or mutated GFP-Polη constructs were irradiated with UVC (15 J m^{-2}) and further incubated for 24 h. The proportions of GFP-Polη expressing cells with more than 30 foci were determined. Data represent means ± SEM from three independent experiments.

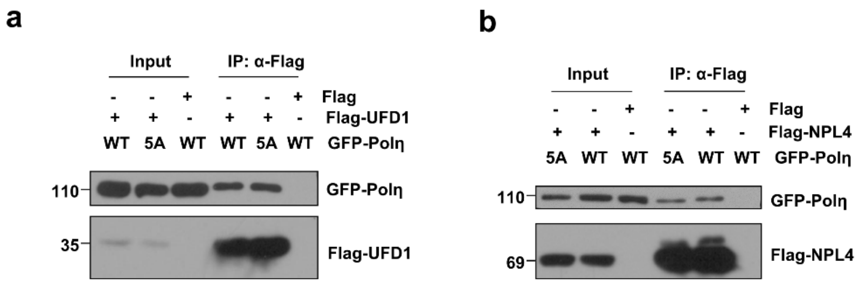

Supplementary Figure 4. WT and 5A Polη manifest similar associations with UFD1 and NPL4, two core adaptor factors of p97. (**a**) Flag empty vector or Flag-UFD1 and GFP-Polη (WT or 5A) were transfected into 293T cells. The lysates were immunopricipitated using anti-Flag M2 agarose. The immunoprecipitates were blotting with anti-GFP and anti-Flag antibodies. The input included 2% of the cell lysate used. (**b**) Flag empty vector or Flag-NPL4 and GFP-Polη (WT or 5A) were transfected into 293T cells. The lysates were immunoprecipitated and analyzed as in (**a**).

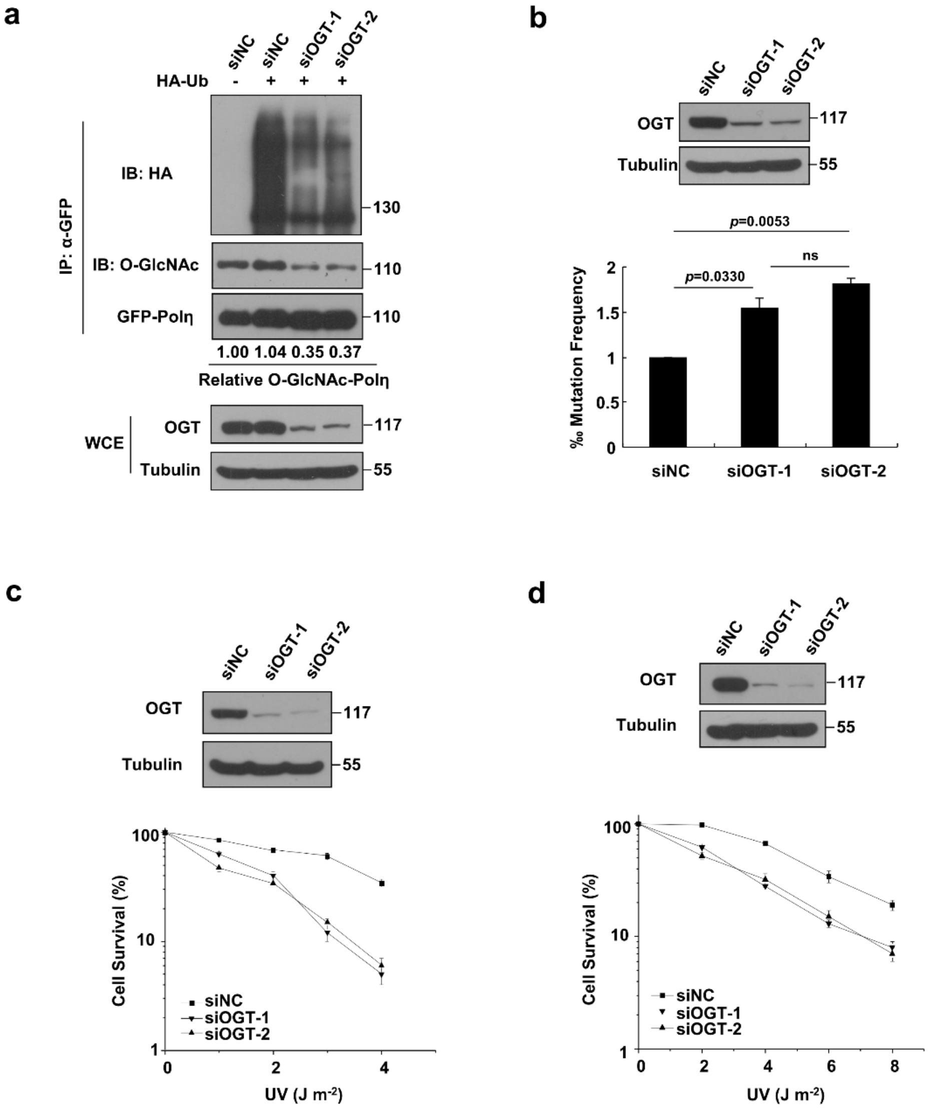

Supplementary Figure 5. OGT affects cellular response to UV radiation. (**a**) GFP-Polη and HA-Ub or HA-vector were transfected into siOGT-treated 293T cells. The cell lysates were immunoprecipitated using GFP-Trap A. The immunoprecipitates were analyzed via western blot using antibodies against GFP, HA and O-GlcNAc. The protein levels of OGT were detected by immunoblotting. WCE: whole cell lysate. Tubulin: loading control. (**b**) Mutation frequency in damaged (400 J m^{-2} UVC) *supF* plasmid was determined as described in “Methods”. ns, not significant. The protein levels of OGT were detected by immunoblotting. Tubulin: loading control. (**c**) & (**d**) U2OS (**c**) or MRC5 (**d**) cells transfected with siOGT or siNC oligos were irradiated with indicated doses of UVC and further incubated in medium supplemented with 0.4 mM caffeine for 7-10 days. The number of cell clones was determined. Surviving fraction was expressed as a percentage of mock-treated

cells. Experiment was repeated three times, giving similar results. The representative curve is shown. Error bar: s.d., n = 3. The protein levels of OGT were detected by immunoblotting. Tubulin: loading control.

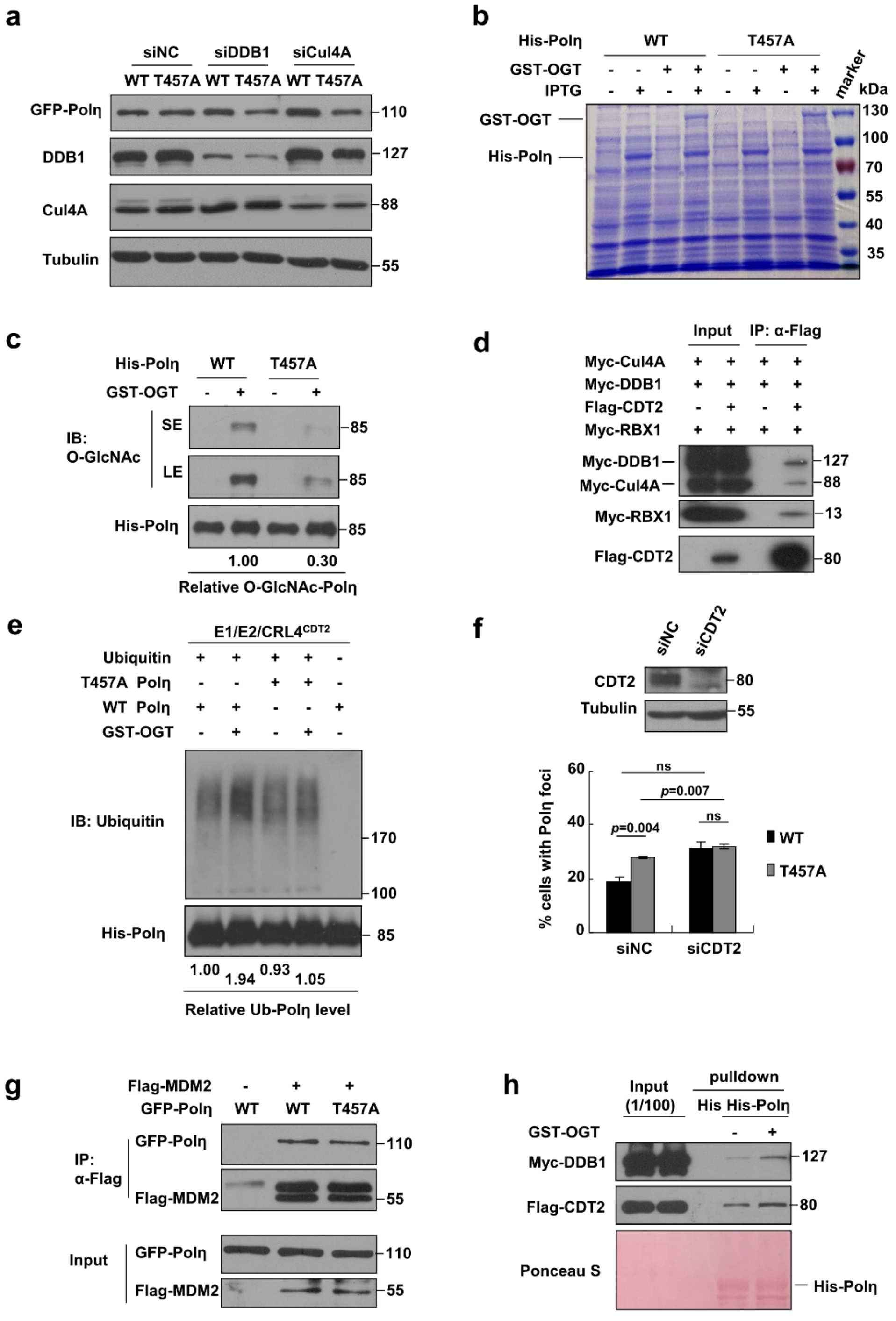

Supplementary Figure 6. T457A mutation-induced delayed Polη removal from replication fork is regulated by CRL4^{CDT2} E3 ligase complex. (**a**) XP30RO cells stably expressing WT or T457A GFP-Polη were transfected with siDDB1, siCul4A or siNC oligos. 72 h later the cells were harvested and the protein levels of Polη, DDB1 and Cul4A were analyzed by western blot. Tubulin is a loading control. (**b**) & (**c**) His-Polη were co-transformed with GST-OGT or empty vector into *E.coli* Transetta (DE3) cells. Protein

expressions after IPTG (0.4 mM) induction were examined via SDS-PAGE followed by coomassie blue staining (**b**). His-Polη proteins were purified and analyzed by western blot with anti-O-GlcNAc and anti-His antibodies (**c**). SE: short exposure; LE: long exposure. (**d**) The CRL4^{CDT2} E3 ligase components were co-expressed in 293T cells and immunoprecipitated using anti-Flag M2 agarose. The immunoprecipitates were eluted by Flag peptide and verified by western blot using anit-Flag and anti-Myc antibodies. (**e**) Puried His-Polη proteins from (**c**) and eluted E3 ligase from (**d**) were incubated with E1, E2 and ubiquitin as indicated. After *in vitro* ubiquitination, the reaction mixtures were resolved by SDS-PAGE and analyzed by inmmunobloting with ubiquitin and Polη antibodies. (**f**) U2OS cells treated with siCDT2 or siNC oligos were transfected with WT or T457A GFP-Polη and UVC (15 J m^{-2}) irradiated 24 h later. The cells were fixed and the proportion of GFP-Polη expressing cells with more than 30 foci was determined. All experiments were carried out in triplicate. Error bar: s.e.m. ns, not significant. (**g**) Flag empty vector or Flag-MDM2 and GFP-Polη (WT or T457A) were transfected into 293T cells. The lysates were immunoprecipitated using anti-Flag M2 agarose. The immunoprecipitates and inputs were examined via western blot using anti-GFP and anti-Flag antibodies. (**h**) Puried His-Polη proteins from (**c**) were incubated with cell lysates expressing Myc-DDB1 and Flag-CDT2. The bound proteins were resolved by SDS-PAGE and analyzed by immunoblotting with anti-Myc and anti-Flag antibodies.

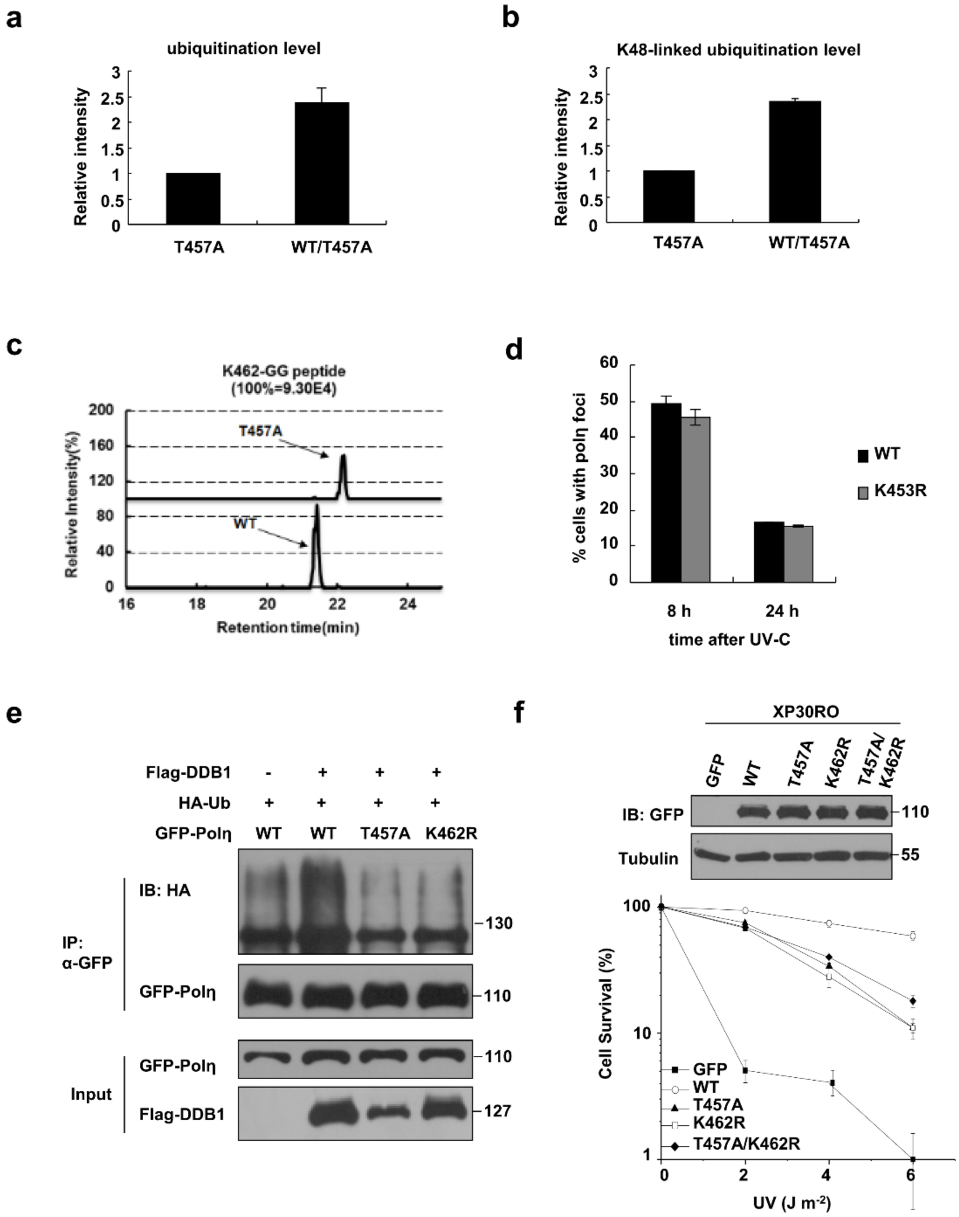

Supplementary Figure 7. K462 in Polη is a potential CRL4^{CDT2} E3 ligase target site, whose ubiquitination is inhibited by T457A mutation. 293T cells transfected with Flag empty vector, WT or T457A Flag-Polη and HA-Ub were immunoprecipitated with anti-Flag agarose in a denatured condition followed by quantitative mass spectrometry analysis as described in “Methods”. Error bar: s.e.m. The levels of general ubiquitination (**a**) and K48-linked ubiquitination (**b**) of WT and T457A Polη were compared. (**c**) K462 residue adjacent to T457 was detected to be ubiquitinated and the relative intensity of K462-GG peptide in WT and T457A Polη was measured. (**d**) U2OS cells transfected with WT or K453R GFP-Polη constructs were UVC (15 J m^{-2}) irradiated and incubated for 8 h and 24

h. The cells were fixed and the proportions of GFP-Polη expressing cells with more than 30 foci were determined by counting at least 200 cells from three independent experiments. Error bar: s.e.m. (**e**) 293T cells were transfected with WT or mutated (T457A, and K462R) GFP-Polη, HA-Ub and Flag-DDB1. The lysates were immunoprecipitated using anti-GFP agarose. The immunoprecipitates were immunoblotted with anti-GFP, anti-HA and anti-Flag antibodies. (**f**) XP30RO cells stably expressing GFP, WT, T457A, K462R or T457A/K462R GFP-Polη were irradiated with indicated doses of UVC and further incubated in medium supplemented with 0.4 mM caffeine for 7-10 days. The number of cell clones was determined. Surviving fraction was expressed as a percentage of mock-treated cells. Experiment was repeated three times, giving similar results. The representative curve is shown. Error bar: s.d., n = 3. The protein levels of GFP-Polη were analyzed by western blot. Tubulin is a loading control.

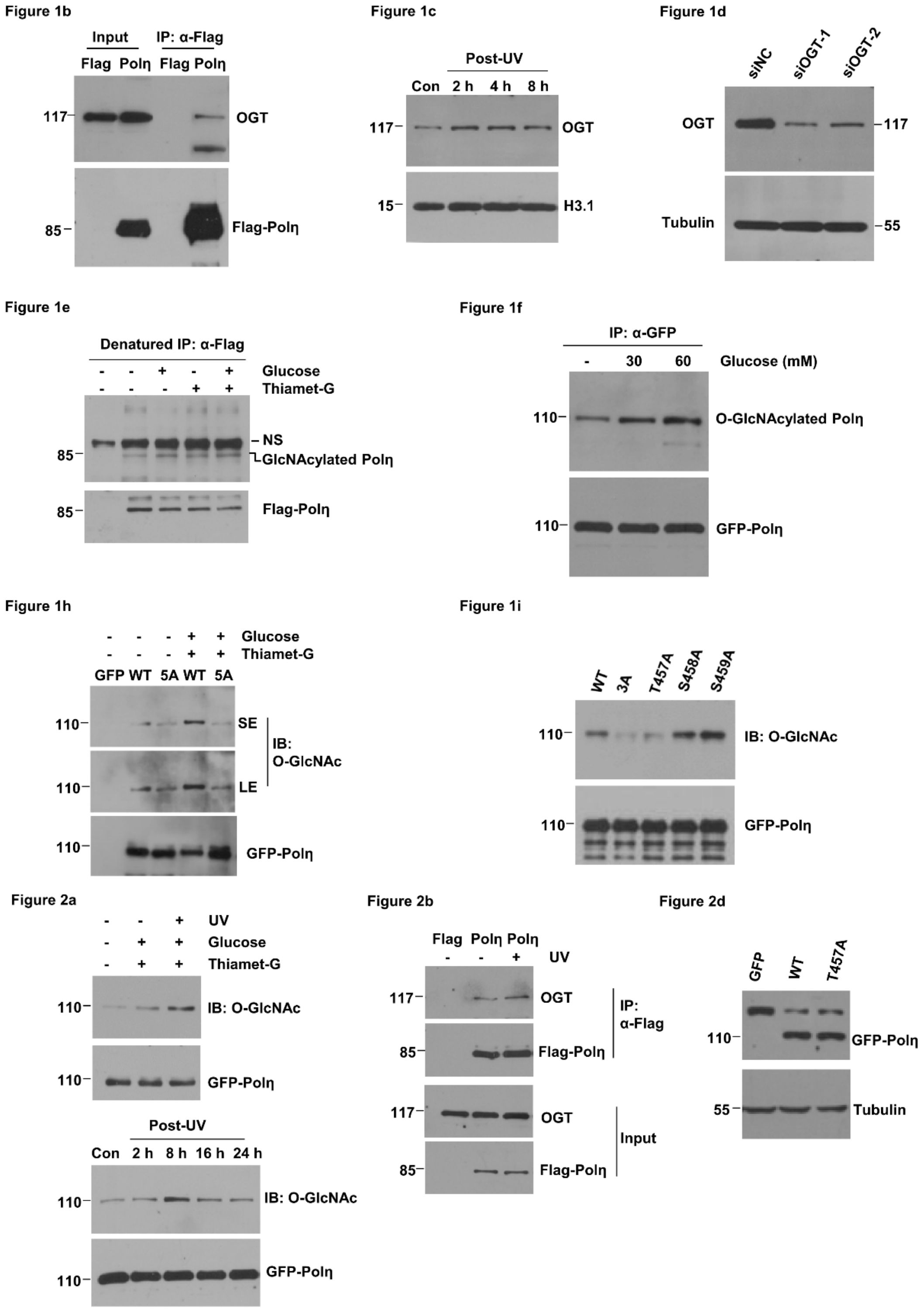

Supplementary Figure 8. Uncropped immunoblots of main Figures.

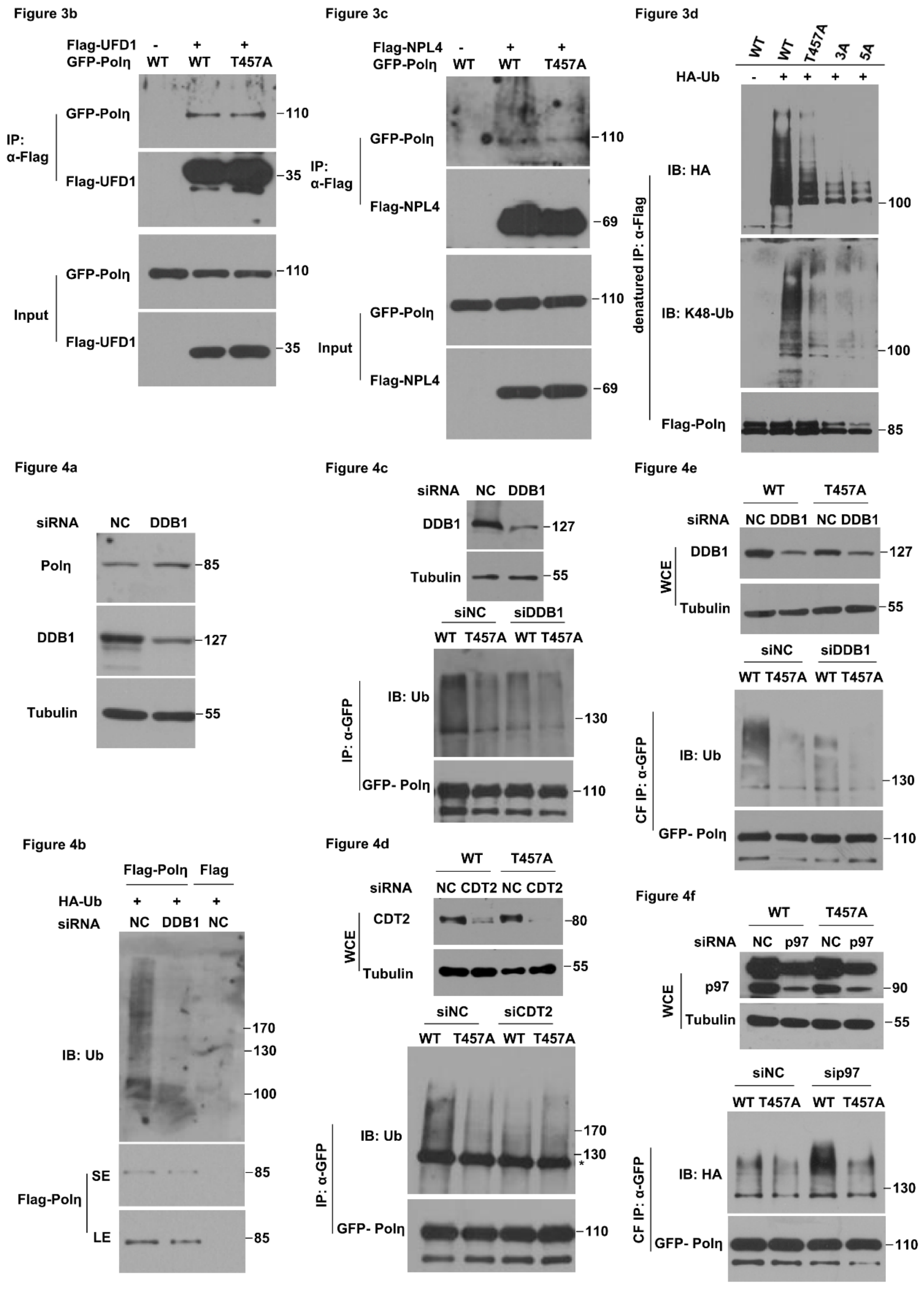

Supplementary Figure 9. Uncropped immunoblots of main Figures.

Figure 4g

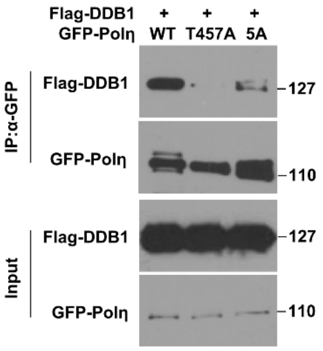

Figure 4h

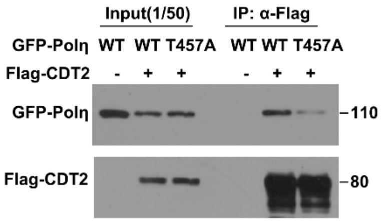

Figure 5e

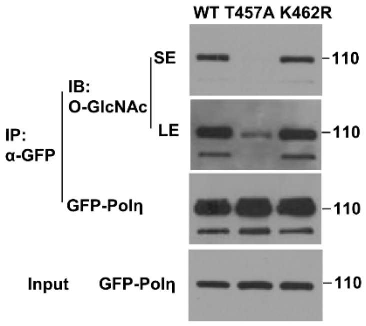

Figure 5c

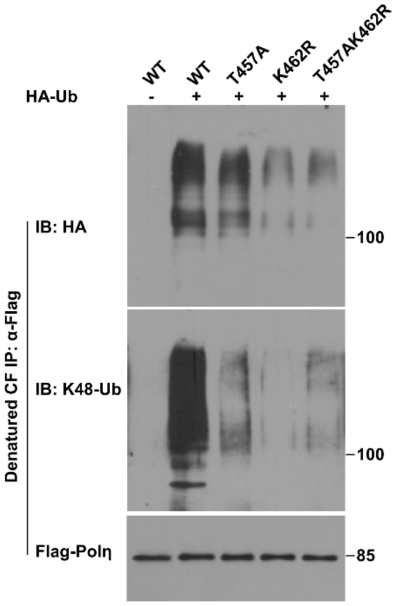

Figure 5f

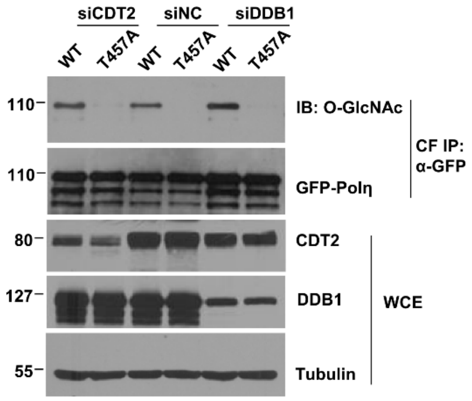

Figure 6a

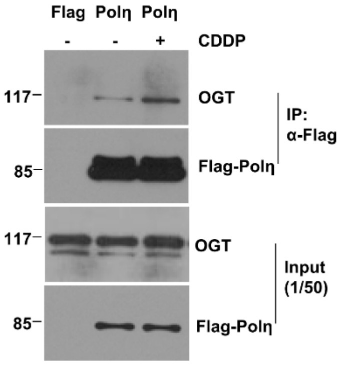

Figure 6b

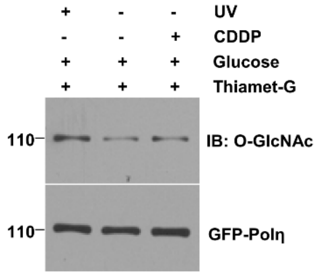

Supplementary Figure 10. Uncropped immunoblots of main Figures.
